# A mechanosensitive Ca^2+^ channel activity is dependent on the developmental regulator DEK1

**DOI:** 10.1038/s41467-017-00878-w

**Published:** 2017-10-18

**Authors:** Daniel Tran, Roberta Galletti, Enrique D. Neumann, Annick Dubois, Reza Sharif-Naeini, Anja Geitmann, Jean-Marie Frachisse, Olivier Hamant, Gwyneth C. Ingram

**Affiliations:** 1 Laboratoire Reproduction et Développement des Plantes, Université de Lyon, ENS de Lyon, UCB Lyon 1, CNRS, INRA, F-69342 Lyon, France; 2 Institute for Integrative Biology of the Cell (I2BC), CEA, CNRS, Université Paris-Sud, Sciences Plant Saclay, Avenue de la Terrasse, 91198 Gif sur Yvette Cedex, France; 3 Department of Physiology and Cell Information Systems, McGill University, Montreal, Québec Canada H3G-0B1; 4 Department of Plant Science, McGill University, Ste-Anne-de-Bellevue, Montreal, Québec Canada H9X3V9

## Abstract

Responses of cells to mechanical stress are thought to be critical in coordinating growth and development. Consistent with this idea, mechanically activated channels play important roles in animal development. For example, the PIEZO1 channel controls cell division and epithelial-layer integrity and is necessary for vascular development in mammals. In plants, the actual contribution of mechanoperception to development remains questionable because very few putative mechanosensors have been identified and the phenotypes of the corresponding mutants are rather mild. Here, we show that the *Arabidopsis* Defective Kernel 1 (DEK1) protein, which is essential for development beyond early embryogenesis, is associated with a mechanically activated Ca^2+^ current *in planta*, suggesting that perception of mechanical stress plays a critical role in plant development.

## Abstract (web summary)

A rise in cytoplasmic Ca^2+^ concentration is a well-described response of plant cells to mechanical stimulation. Here the authors show that the DEK1 protein, which is essential for epidermis specification and development in plants, is required for triggering a mechanically-activated Ca^2+^ channel.

## Introduction

Multicellular development is dependent upon cell−cell communication. Recent research has highlighted the fact that, in addition to chemical signals, the perception of mechanical stress, at both the cell and tissue level, is a key factor underlying growth coordination and morphogenesis (e.g., ref. ^1^). However, while in animal systems mechanosensors with important roles in development have been identified^2–9^, the molecular basis for mechanoperception in plants remains enigmatic. As in animals, plants respond to mechanical stimuli by an elevation in cytoplasmic Ca^2+^ ^10–12^. Based on the knowledge of plasma-membrane mechanosensing in animal systems, the most probable trigger for Ca^2+^ release from internal cellular compartments (Ca^2+^-induced calcium release) is either the opening of plasma membrane-localized mechanosensitive Ca^2+^ permeable channels, or the opening of voltage-dependent Ca^2+^ channels in response to changes in membrane potential caused by mechanosensitive channels permeable to other ions^12^. Recent research in *Arabidopsis* has led to the identification of several plasma membrane-localized mechanosensitive ion channels. These include proteins similar to the bacterial mechanosensitive channel of small conductance (the MSL family^13–16^), the MCA1 protein that rescues the yeast Ca^2+^ channel mutant *mid1*, and its homolog MCA2^17–20^. In addition, the membrane protein OSCA1 forms a hyperosmolarity-gated Ca^2+^ permeable channel required for osmosensing in *Arabidopsis* ^21^. However, although the MSL8 protein has recently been shown to be required for pollen grains to survive rapid rehydration during fertilization^14^, the very mild developmental phenotypes in single and multiple mutants of genes encoding the channels described above, suggests that they are unlikely to play a major role in mechanosensing during development. Furthermore, these proteins have not conclusively been shown to be responsible for any of the mechanosensitive Ca^2+^ currents which have been detected and extensively described by electrophysiologists over the past few decades *in planta* ^20, 22–26^. Since genes encoding voltage-sensitive Ca^2+^ channels similar to those identified in animal systems have not been found in plant genomes, it has been proposed that plants may have evolved novel systems for mediating mechanosensitive Ca^2+^ fluxes at the plasma membrane to control development (reviewed in ref. ^12^).

The Defective Kernel 1 (DEK1) protein is encoded by a highly conserved uni-gene found in all multicellular plant genomes sequenced so far, and it is absolutely required for both embryonic and post-embryonic development in angiosperms^27–31^. Null *dek1* mutant embryos do not develop beyond the early globular stage. When plants with reduced DEK1 activity can be obtained, they show major developmental defects, notably in epidermal differentiation and adhesion^30, 32, 33^.

The DEK1 protein contains multiple predicted transmembrane (TM) spans interrupted by a loop, and an intracellular tail including a linker domain and a C-terminal domain showing similarity to animal calpains, a class of Ca^2+^-dependent cysteine proteases^34^. DEK1 localizes to the plasma membrane and to internal compartments^32, 34^. In their original model of DEK1, Lid et al.^34^ predicted the presence of 21 TM domains with an extracellular localization for the loop and cytoplasmic localization (subsequently confirmed *in planta*) for the C-terminus. However, in a more recent analysis, Kumar et al.^35^ proposed a consensus model with 23 TM domains and with a cytoplasmic localization for the loop. Structure-function studies have shown that the cytosolic CALPAIN domain of the protein can, alone, complement the embryo lethality of *dek1* mutants suggesting that this domain, which is removed from the rest of the protein by an autolytic cleavage event, represents an active form of the DEK1 protein^32^. Consistent with this scenario, and with a role for DEK1 in maintaining epidermal integrity, overexpression of the CALPAIN domain of DEK1 leads to thickening of the outer epidermal cell wall in leaves, and increased deposition of pectins^36^, which are important for cell adhesion (reviewed in ref. ^37^). The epidermis is thought to be under tension during much of plant growth^38^. One plausible function of DEK1 could, therefore, be to perceive and respond to this tension to coordinate epidermal development and maintain epidermal integrity. As the epidermis plays a critical role in organ growth in plants (e.g., refs. ^39–42^), this, in turn, has major implications for plant development as a whole.

The activity of the CALPAIN domain has been shown to be Ca^2+^-dependent in vitro^43^. These findings are in line with results in animal systems where the activation of cytoplasmic calpains is associated with the Ca^2+^-dependent autolytic-removal of an N-terminal extension, which in some cases can inhibit calpain activity^44–46^. Because calpains have been shown to act downstream of mechanosensitive channels such as PIEZO in animals^4^, we tested the hypothesis, and demonstrated, that the TM domain of DEK1 is associated with Ca^2+^ dependent mechanoperception at the plasma membrane in plants.

## Results

### A rapidly mechanically activated plasma membrane Ca^2+^ channel

Combining genetic and electrophysiological approaches, the *Arabidopsis* MSL9 and MSL10 proteins have been shown to mediate a mechanically activated channel activity in protoplasts derived from wild-type plant root cells^13^. Selectivity characterization indicated that anions permeate preferentially through the wild-type MSL channels^13^. Because in this work we used excised membrane patches from callus protoplasts, rather than whole root cell-derived protoplasts, we first investigated whether mechanically activated ion currents due to MSLs and other unidentified channels could also be detected in this material.

**Fig. 1:**
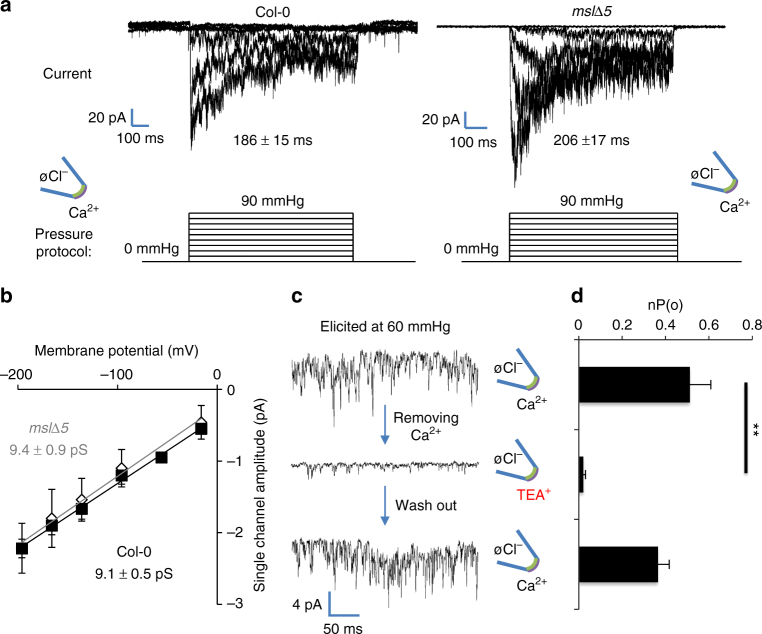
A mechanically activated current permeable to Ca^2+^ is present at the plasma membrane. **a** Representative membrane patches from Col-0 (*left*) and an *msl* quintuple mutant *msl4*;*msl5*;*msl6*;*msl9*;*msl10* (*mslΔ5*, *right*), exposed to increased positive pressure steps in an outside-out patch configuration show a rapidly activated, rapidly inactivated current in ionic conditions favorable for Ca^2+^ current recording. We have named the channel responsible for this activity the RMA channel (see text). Time constants are means ± SE (*n* = 6); **b** Under the same conditions, single channel I/V curves show similar RMA channel conductance in Col-0 (*solid square*) and in the mslΔ5 mutant (*open square*). Values are means ± SE (*n* = 6); **c** Representative single channel recordings showing that the RMA current is reversibly eliminated by exchanging Ca^2+^ ions with non-permeant TEA^+^ ions. **d** Open probability (for n channels,﻿ (nP(o)) is severely decreased in TEA^+^ bath solution. A paired *t*-test was used to compare means (**P < 0.01). Values are means ± SE. For all experiments, the membrane potential was held at −196 mV. Ionic conditions are described in the Methods section

Reverse transcription quantitative-PCR (RT-qPCR) analysis confirmed the expression of *MSL9* and *MSL10* in wild-type (Columbia-0) callus tissue (Supplementary Fig. 1a). When Cl^−^ is provided at the cytosolic face of plasma membrane patches, a mechanically activated channel with a high conductance (46 ± 1.4 pS; ± indicates standard deviation, *n* = 6) is elicited upon application of positive pressure using a high-speed pressure clamp, as previously described in root protoplasts (Supplementary Fig. 1b)^13^. This activity, which is mainly due to MSL9 and MSL10, was completely abolished in protoplasts obtained from quintuple *msl4 msl5 msl6 msl9 msl10 (mslΔ5)* mutant callus (Supplementary Fig. 1c). In addition, to MSL activity, we observed a rapidly activated-inactivated current with a smaller conductance, elicited immediately after the pressure increase (Supplementary Fig. 1b). Unlike the situation for the MSL current, removing Cl^−^ from the cytosolic face of the membrane did not modify this rapid current (Fig. 1a, Supplementary Fig. 1b). The rapid current was still present in protoplasts derived from *mslΔ5* mutant callus with or without Cl^−^ ions at the cytosolic face (Fig. 1a, Supplementary Fig. 1c). In addition this mechanically activated channel showed a similar conductance to that seen in Col-0 callus (Fig. 1b), with a reversal potential at positive voltage (*E*_rev_WT = 47.5 ± 5.4 mV and *E*_rev_ *msl∆5* = 60.6 ± 11.5 mV; ± indicates standard deviation, *n* = 6) suggesting that this rapid current is most likely mediated by Ca^2+^ influx (dominant cation gradient, *E*_Ca_2+=128.7 mV). Consistent with this hypothesis, we found that it could be reversibly eliminated by replacing Ca^2+^ in the bath solution with the larger TEA^+^ cation (Fig. 1c, d). The pressure required to activate the current was higher than that required to activate MSL channels, suggesting distinct activation thresholds for these channels (Supplementary Fig. 1b, c). Thus, we can distinguish at least two independent mechanically activated channel activities in callus protoplasts: one dependent on MSLs activity and the second due to an unidentified Ca^2+^ channel which we called the rapid mechanically activated (RMA) channel.

**Fig. 2:**
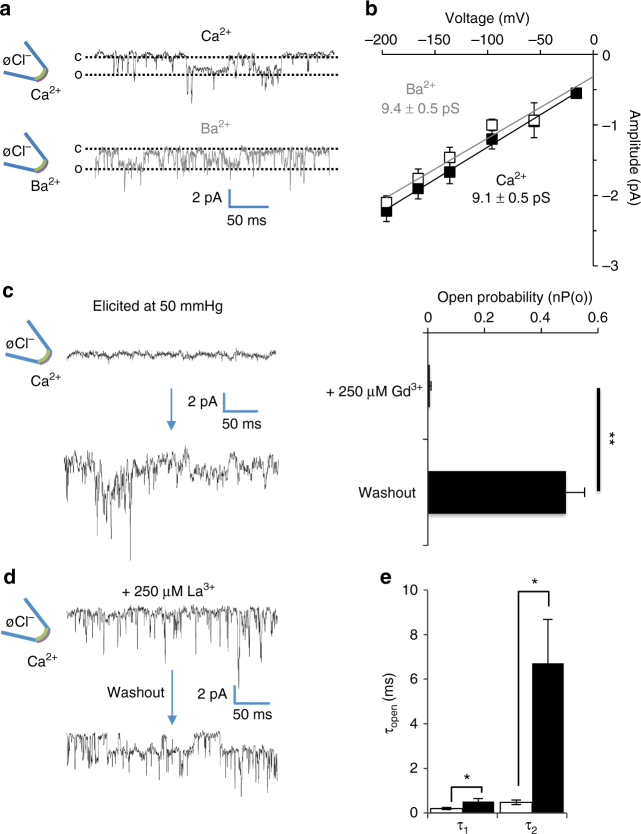
The mechanically activated RMA current is preferentially permeable to Ca^2+^ and affected by both the mechanosensitive channel inhibitor Gd^3+^ and the calcium channel blocker La^3+^. **a** Representative single channel recordings in response to positive pressure, in an outside-out patch configuration with Ca^2+^ (*top*) or Ba^2+^ (*bottom*) as the permeant bivalent cation in the bath. The *dotted lines* indicate the open (*O*) and closed (*C*) channel state; **b** Single channel I/V curves show similar conductance with permeant cations Ca^2+^ (*solid square*) and Ba^2+^ (*open square*). Values are means ± SE (*n* = 6); **c** The mechanically activated RMA current is inhibited by Gd^3+^ and restored after wash-out (*left, n* = 6) and the corresponding open probability (nP(o), *right*). Values are means ± SE; **d** Representative recording showing the effect of La^3+^ (*left*, *n* = 6) on the RMA current; **e** La^3+^ ions affects the open state of the RMA channel and reducing the mean open time. *Bars* represent the two time constant τ_1_ and τ_2_ of the open state with (*white bars*) or without (*black bars*) La^3+^. A paired *t-*test was used to compare means (*P* < 0.01). E_rev_(Ca^2+^) = 47.5 ± 5.4 mV; E_rev_(Ba^2+^) = 36.5 ± 6.1 mV. Values are means ± SE (open events, *n* ≥ 100). For all experiments, the membrane potential was held at −196 mV. Ionic conditions are described in the Methods section

To understand the nature of the RMA-mediated current we tested a range of pipette and bath solutions. The RMA channel conductance is unaffected when Ca^2+^ ions are replaced with Ba^2+^ ions in the bath solution (Fig. 2a, b) suggesting that the RMA channel is similarly permeant to divalent Ca^2+^ and Ba^2+^ ions, as is classically found for Ca^2+^ channels. To test whether the RMA channel shows characteristics previously described for mechanically activated channels in plants, we investigated the effect of Gd^3+^ ions, which are known to affect mechanically activated channel activity in both animal and plant systems^23, 47–52^. RMA activity was almost totally blocked by the presence of Gd^3+^ ions, and activity could subsequently be restored by washing out Gd^3+^ (Fig. 2c). The presence of La^3+^ ions, a known blocker of Ca^2+^ channels^53–55^ strongly reduced the mean open time of the RMA channel (Fig. 2d, e) but did not modify the channel conductance. We conclude that the RMA channel activity fulfils the key criteria previously attributed to mechanically sensitive Ca^2+^ channel activities recorded at the plant plasma membrane.

### DEK1 trans-membrane domains are required for RMA activation

Loss of DEK1 activity in *Arabidopsis* leads to early embryo lethality (Supplementary Fig. 2a)^29, 30^. By expressing the cytoplasmically localized CALPAIN domain under a constitutive promoter (the *RPS5A* promoter) in *dek1* mutant alleles^32^, we could restore post-embryonic growth and thus investigate the contribution of the DEK1 TM domain to RMA activation. This was done in both the *dek1-2* mutant background (which has a T-DNA insertion toward the beginning of the TM encoding regions (Supplementary Fig. 2b, c)^32^) and in the *dek1-3* mutant background (which contains a T-DNA downstream of the TM encoding regions of the *DEK1* gene (Supplementary Fig. 2b, c)^32^). Calli were generated from both *dek1-2* CALPAIN-OE and *dek1-3* CALPAIN-OE lines and genotyped (Supplementary Fig. 3a−c). In callus from CALPAIN complemented *dek1-3* mutants (*dek1-3 CALPAIN-OE*), we detected wild-type levels of the transcript corresponding to the endogenous TM-span encoding region of *DEK1* (Supplementary Figs. 2c, 4a, b). This allele therefore has the potential to encode a truncated version of the DEK1 protein, with an intact set of TM spans. However, in callus from complemented *dek1-2* mutants (*dek1-2 CALPAIN-OE*), the TM domain-encoding region of the *DEK1* gene is physically disrupted (Supplementary Figs. 2c, 4a, c) and an aberrant transcript is produced downstream of the T-DNA insertion (Supplementary Fig. 4b). This transcript is unlikely to be translated to produce a functional truncated protein as the *dek1-2* allele shows a null phenotype. Overexpression of the CALPAIN transcript was detected in both backgrounds (Supplementary Fig. 4b). We also generated an antibody against the CALPAIN domain and verified overexpression at the protein level (Supplementary Figs. 5a, b, 6).

**Fig. 3:**
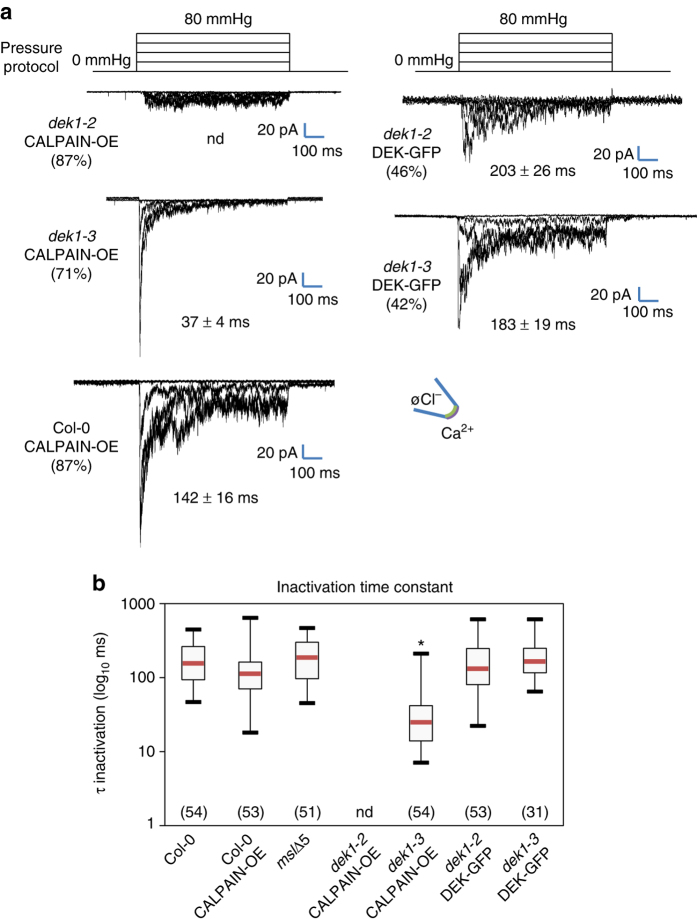
RMA activation depends on the trans-membrane region of the essential DEK1 protein. **a** Mechanosensitive current kinetics in response to pulse pressure (as illustrated) in *dek1-2* CALPAIN-OE, *dek1-3* CALPAIN-OE, Col-0 CALPAIN-OE, *dek1-2* DEK-GFP and *dek1-3* DEK-GFP. Time constants are means ± SE (*n* = 6); **b** Inactivation times are fitted with an exponential function, and time constants (τ_inact_) are represented in a box plot distribution for different lines. Number in *brackets* represent the number of time constants. *significantly different from Col-0 line, Anova on ranks (*P* < 0.05). For all experiments the membrane potential was held at −196 mV. Ionic conditions are described in the Methods section

Because the *dek1-2 CALPAIN-OE* background cannot produce the intact N-terminal region of DEK1, we next tested the potential effect of DEK1 TM domain disruption on the RMA channel activity. In the majority of responsive patches obtained from *dek1-2 CALPAIN-OE* calli we observed a complete absence of the RMA current upon membrane stretching (Fig. 3a, Supplementary Fig. 7). Only a residual current was detectable in most patches of this background. In patches from *dek1-3 CALPAIN-OE* calli the RMA channel activity was still detectable, but showed 6–8 times more rapid inactivation kinetics (Fig. 3a, b, Supplementary Fig. 7) compared to those observed in callus from Col-0 plants.

The production of viable *dek1* mutant plants, as mentioned before, requires the expression of at least the CALPAIN domain of DEK1. In our lines, the CALPAIN domain is overexpressed in the callus system at the RNA level (Supplementary Fig. 4). To confirm that the loss of the RMA channel activity in the *dek1-2 CALPAIN-OE* background is not a consequence of CALPAIN overexpression, we generated calli from Col-0 plants over-expressing the CALPAIN domain (Supplementary Figs. 4d and 5)^18^. This material was submitted to electrophysiological analysis. The RMA current was still present in patches from this material, and behaved similarly to the current in Col-0 patches. (Fig. 3a, b, Supplementary Fig. 7). Therefore, we conclude that the CALPAIN domain overexpression *per se* is not responsible for the loss of RMA channel activity in the *dek1-2 CALPAIN-OE* background.

### Loss of the DEK1 TM domains does not affect MSL activity

Next, we investigated whether the removal of the DEK1 TM domains could cause a perturbation of other mechanoresponsive channel activities at the plasma membrane by testing whether MSL channels can be activated in the *dek1-2 CALPAIN-OE* line. We found that the MSL activity in *dek1-2 CALPAIN-OE* protoplasts was still detectable upon membrane stretching, and its characteristics were comparable to that of Col-0 (Supplementary Fig. 8). Thus, loss of the DEK1 TM domains leads to a specific perturbation of the mechanosensitive RMA channel activity in our system.

### DEK1 function influences root growth inhibition by Gd^3+^ ions

Based on the mild developmental defects observed in the *dek1* CALPAIN-OE lines^32, 56^, the CALPAIN domain of DEK1 is likely the “active” domain of the protein. The DEK1 CALPAIN domain is autolytically cleaved from the full length DEK1 protein^32^, and its activity has been shown to be Ca^2+^ dependent *in vitro* ^43^. Because the release of the CALPAIN domain upon DEK1 cleavage could be triggered by Ca2+ influx via the RMA channel, blocking the RMA channel might affect development in wild-type plants, but would have a reduced impact in the *dek1-2* CALPAIN-OE line, where the expression of a “pre-cleaved” version of the DEK1 CALPAIN would partially bypass the need for the RMA channel activity. Considering the sensitivity of the RMA current to Gd^3+^, and the absence of this current in CALPAIN complemented *dek1-2* mutants we tested whether CALPAIN complemented *dek1-2* mutants might show such reduced sensitivity to Gd^3+^. Gd^3+^ dramatically inhibits *Arabidopsis* root growth^57^. We generated a dose sensitivity curve of wild-type Col-0 seedlings to concentrations of Gd^3+^ between 0 and 200 μM (Supplementary Fig. 9). We found strong growth inhibition (40–50%) in wild-type seedlings even at the lowest concentrations tested (30 and 60 μM) (Supplementary Fig. 9). This inhibition was alleviated in CALPAIN complemented *dek1-2* plants (Supplementary Fig. 10), formally relating DEK1 function to Gd^3+^ sensitivity *in planta*.

### Reintroducing full-length DEK1 restores RMA current

We hypothesize that the loss of RMA current observed in *dek1-2 CALPAIN-OE* callus is caused by the absence of an intact DEK1 transmembrane region. We propose that in the *dek1-3 CALPAIN-OE* plants a truncated DEK1 protein, which contains the DEK1 transmembrane spans but which cannot confer normal inactivation kinetics on the RMA current is produced (Supplementary Fig. 2c). To test both these hypotheses we investigated the RMA channel activity in both the previously published and characterized *dek1-3* line complemented with the full length GFP-tagged DEK1 protein expressed under the *RPS5A* promoter (*dek1-3 DEK1*)^30^, and in a *dek1-2* mutant background into which the same, complementing full length DEK1-encoding construct had been introduced by crossing (*dek1-2 DEK1*). In the majority of patches obtained from these lines, we recorded an RMA channel activity with inactivation kinetics and current amplitude resembling those of wild-type plants (Fig. 3a, b; Supplementary Fig. 7). The properties of this restored current were tested in the complemented *dek1-2 DEK1* mutant callus and found to be strikingly similar to those of the RMA current (Supplementary Fig. 11). Our results show that reintroducing the full length DEK1 into *dek1* mutant backgrounds leads to the restoration of an RMA current similar to that observed in wild-type callus.

## Discussion

In summary, we show that the mechanosensitive activity of a Ca^2+^-permeable channel present in the plasma membrane of *Arabidopsis* callus-derived protoplast requires the TM domains of the DEK1 protein. It has previously been demonstrated that the CALPAIN domain of DEK1 is released from the plasma membrane by an autolytic cleavage event^32^, and that the CALPAIN activity is enhanced by Ca^2+^ ions^43^. We therefore propose a model in which DEK1 activity leads to transient elevation of cytoplasmic Ca^2+^ concentration during mechanical stimulation, which is locally transduced by autolytic cleavage (and thus activation) of the CALPAIN domain^32^. Based on the phenotype of lines with reduced DEK1 activity, this mechanotransduction pathway is likely required for the maintenance of cell−cell contacts and epidermis integrity^33^, consistent with embryo lethality in loss of function alleles. In turn, DEK1-dependent epidermis integrity is required for the propagation of mechanical signals between neighboring cells, in a feedback loop. Given the absolute requirement for DEK1 in both coherent embryogenesis and post-embryonic growth^29–31, 33^ (summarized in Supplementary Fig. 12), our work suggests a key contribution of mechanoperception to plant development. The absence of cell migration in plant tissues may explain why the perception of tension between adjacent cells plays such an essential role in development in this kingdom.

This study, together with previously published results from both our work and the work of others^32, 56^ supports the idea that the CALPAIN domain of DEK1 is the effector component of the protein in terms of mechanotransduction. Whether the transmembrane portion of DEK1 forms a mechanically activated Ca^2+^ channel per se, or it is the mechano-sensor associated with an independent Ca^2+^ channel remains to be determined. However, the published Ca^2+^ dependence of CALPAIN catalytic activity, combined with our electrophysiological results and the fact that CALPAIN complemented *dek1* null mutants develop relatively normally, suggests that once activated by cleavage, the CALPAIN domain of DEK1 can respond to changes in cytoplasmic Ca^2+^ levels mediated by other Ca^2+^ channels. These could include proteins such as MCA1 and MCA2, or potentially the plant PIEZO protein, which has yet to be functionally characterized. The presence of such channels is consistent with our finding that CALPAIN complemented *dek1-2* plants retain some Gd^3+^ sensitivity *in planta*.

Interestingly, intracellularly localized calpains have been proposed to act downstream of mechanosensitive ion channels in animals, to regulate a variety of cellular processes, including cell-to-cell adhesion^58–61^. In this context, the association of a calpain protease with a domain influencing a mechanosensitive Ca^2+^ channel activity in the membranes of multicellular plants may reflect evolutive convergence between these two kingdoms.

In animals, the characterization of mechanosensitive channels Piezo1 and Piezo2 has revealed novel roles of mechanoperception in development and physiology. For instance, respiration in lungs relies on Piezo2-expressing sensory neurons, which use mechanical signals to sense airway stretching^62^. Axon growth and trajectory was also shown to depend on Piezo1-dependent perception of the mechanical environment of neurons^63^. The association of DEK1, a key developmental regulator, with a mechanosensitive channel activity paves the way to the identification of new roles of mechanical signals in plant development.

## Methods

### Plant material

*Arabidopsis* (*Arabidopsis thaliana*) Columbia-0 (Col-0) wild-type seeds were obtained from Nottingham *Arabidopsis* Stock Centre (NASC, School of Biosciences, University of Nottingham, United Kingdom). Plants expressing the *pRPS5A:CALPAIN-HIS* in a wild-type background (*CALPAIN-OE*), *pRPS5A:CALPAIN-GFP*, or *pRPS5A:DEK1* in the *dek1-3* mutant background and *pRPS5A:CALPAIN-GFP* in the *dek1-2* background have been previously described^18, 30^. The *pRPS5A:DEK1* construct was transferred from the *dek1-3* to the *dek1-2* background by crossing, and plants were genotyped as described in Supplementary Fig. 3.

### Plant and callus growth conditions

For *in vitro* cultures, seeds were surface-sterilized with chlorine gas, sown on square plates, and stratified for 2 or 3 days in the dark at 4 °C. After stratification, seeds were germinated in a growth chamber under a 16-h light/8-h dark cycle at 21 °C.

### Callus generation

Surface-sterilized seeds were sown on “initiation medium” containing 4.3 g/L Murashige and Skoog salts (MS, Sigma-Aldrich), 2% sucrose, 10 mg/L myo-inositol, 100 µg/L nicotinic acid, 1 mg/L thiamine-HCl, 100 µg/L pyridoxine-HCl, 400 µg/L glycine, 0.23 µM kinetin, 4.5 µM 2,4-D, 1% Phytagel, (pH 5.7). For callus generation, seeds were cultured in a growth chamber for 15 days. Calli were then transferred onto “maintenance medium” containing 4.3 g/L MS salts (Sigma-Aldrich), 2% sucrose, 10 mg/L myo-inositol, 100 µg/L nicotinic acid, 1 mg/L thiamine-HCl, 100 µg/L pyridoxine-HCl, 400 µg/L glycine, 0.46 µM kinetin, 2.25 µM 2,4-D, 1% phytagel, (pH 5.7), and sub-cultured every 15 days onto fresh “maintenance medium”.

### Protoplasting protocol

Calli were digested for 15 min at 22 °C under hyperosmotic conditions (2 mM CaCl_2_, 2 mM MgCl_2_, 1 mM KCl, 10 mM MESs (pH 5.5), 0.2% cellulysin (Calbochem), 0.2% cellulase RS (Onozuka RS, Yakult Honsha Co.), 0.004% pectolyase Y23 (Kikkoman Corporation), 0.35% bovine serum albumine and mannitol to 600 mOsmol. For enzyme removal, the preparation was washed twice with 2 mM CaCl_2_, 2 mM MgCl_2_, 10 mM MES (pH 5.5), and mannitol to 600 mOsmol. For protoplast release, the preparation was incubated with 2 mM CaCl_2_, 2 mM MgCl_2_, 10 mM MES (pH 5.5), and mannitol to 280 mOsmol. The suspension was filtered through a 50 µm nylon mesh.

### Electrophysiology

Patch-clamp experiments were performed at room temperature with a patch-clamp amplifier (model 200 A, Axon Instruments, Foster City, CA) and a Digidata 1322 A interface (Axon Instruments). Currents were filtered at 5 kHz, digitized at 20 kHz, and analyzed with pCLAMP8.1 and Clampfit 10 software. During patch-clamp recordings, cells were held at a holding potential (corrected from liquid junction potential) of −16 or −6 mV depending on the composition of the pipette solution and pressure was applied with a high speed pressure-clamp system (ALA Scientific Instrument, NY), allowing the application of precise and controlled pressure pulses in the pipette^15, 64^. For Ca^2+^ and Ba^2+^ current recordings, bath solutions contained: 50 mM CaCl_2_ or 50 mM BaCl_2_, respectively, and 5 mM MgCl_2_, 10 MES-Tris (pH 5.6); while pipettes were filled with: 150 mM CsMES, 2 mM MgCl_2_, 5 mM EGTA, 4.2 mM CaCl_2_, and 10 mM Tris-HEPES (pH 7.2), supplemented with 5 mM MgATP. To remove Ca^2+^, a solution containing: 100 mM TeaCl, 5 mM MgCl_2_ and 10 mM MES-Tris (pH 5.6) was used.

For Cl^−^ current recordings, bath solution contained (mM): 50 CaCl_2_, 5 MgCl_2_, 10 MES-Tris (pH 5.6) and pipettes were filled with (mM): 150 CsCl, 2 MgCl_2_, 5 EGTA, 4.2 CaCl_2_, and 10 Tris-HEPES (pH 7.2), supplemented with 5 MgATP. For inhibitor treatments, 0.25 mM LaCl_3_ or 0.25 mM GdCl_3_ were added to the bath solution, osmolarity was adjusted with mannitol to 450 mOsmol for the bath solution and to 460 mOsmol for the pipette solution using an osmometer (Type 15, Löser Meβtechnik). Gigaohm resistance seals between pipettes (pipette resistance, 0.8–1.5 MΩ) (coated with Sylgard (General Electric) pulled from capillaries (Kimax-51, Kimble Glass)) and protoplast membranes were obtained with gentle suction leading to the whole-cell configuration, then excised to an outside-out configuration. The current inactivation kinetics were fitted with a mono-exponential function: F(*t*) = A × e-t/τ + C, where A is the coefficient, τ is the time constant, and C represents the maximum current intensity.

### Genomic DNA extraction and genotyping

Plant callus DNA was extracted using a rapid CTAB isolation technique^65^. *dek1-2* and *dek1-3* mutants were genotyped using the primers shown in Supplementary Table 1 and Supplementary Fig. 3. The following thermal profile was used: 95 °C for 5 min, 35 cycles of 95 °C for 45 s, 60 °C for 45 s, 72 °C for 1 min, and a final extension step of 5 min at 72 °C.

### RNA extraction and quantitative gene expression analysis

Callus material was collected and snap-frozen in liquid nitrogen for gene expression analysis. For each experiment, at least two independent biological replicates were used. Total RNA was extracted using the Spectrum Plant Total RNA Kit (Sigma-Aldrich). Total RNAs were digested with Turbo DNA-free DNAse I (Ambion) according to the manufacturer’s protocol. RNA concentrations were measured with a NanoDrop ND-1000 UV−Vis spectrophotometer (NanoDrop Technologies). One microgram of total RNA was reverse transcribed (RT) using the SuperScript VILO cDNA Synthesis Kit (Invitrogen) according to the manufacturer’s instructions. PCR reactions were performed in optical 96-well plates in the StepOne Plus Real Time PCR System (Applied Biosystems). Five microliters of a 1:10 dilution of cDNA was amplified in 20 µL of reaction mix. The following thermal profile was used: 95 °C for 10 min, 40 cycles of 95 °C for 15 s, 60 °C for 30 s. Amplicon melting curves, were recorded after cycle 40 by heating from 60 to 95 °C with a ramp speed of 1 °C min^−1^. Expression levels of each gene, relative to *EIF4A*, were determined using a modification of the Pfaffl method^33^. Technical triplicates were performed for each biological sample. Primers used for RT-quantitative (q)PCR analysis are shown in Supplementary Table 1.

### Antibody production

Anti-IIaIIb rabbit polyclonal antibodies were generated against a peptide from the predicted catalytic domain (RGDKQFTDQEFPPNC) of the *Arabidopsis* DEK1 protein using the PolyExpress Custom Polyclonal Antibody Production Package Service (GenScript).

### Western blot analysis

Callus material for each genotype was snap-frozen in liquid nitrogen, thoroughly ground in pre-cooled porcelain mortars, and proteins were extracted using an extraction buffer containing 50 mM Tris-HCl pH 7.5, 150 mM NaCl, 1% Nonidet P-40, 10% glycerol, 1% deoxycholate, 1 mM EDTA and 1× protease inhibitor cocktail P9599 (Sigma-Aldrich). Samples were incubated on ice for 1 h, centrifuged for 15 min at 14,000×*g* at 4 °C to remove cell debris, and protein concentrations in supernatants were determined using a Bio-Rad protein assay (Bio-Rad). Equal amounts of proteins were loaded and resolved on 7.5% polyacrylamide/0.1% SDS gels. Proteins were transferred onto nylon membranes using an iBlot II dry blotting system (Invitrogen). Membranes were blocked over night at 4 °C using 1× PBS/0.2% Tween-20 (Sigma-Aldrich) with 5% non-fat milk (Regilait), incubated for 2 h at room temperature with rabbit polyclonal anti-IIaIIb (Genescript) or mouse monoclonal anti-alpha tubulin clone B-5-1-2 (Sigma-Aldrich, catalogue number T5168) primary antibodies at dilutions of 1:1000 and 1:2000, respectively. Secondary horseradish peroxidase-conjugated anti-rabbit and anti-mouse antibodies (Promega, catalogue numbers S3731 and S3721, respectively) were used at a dilution of 1:45,000. Blots were incubated with Super Signal West Femto reagents (Thermo Scientific) according to the manufacturer’s instructions, and then exposed to Super RX film (Fujifilm). Membranes were first incubated with anti-IIaIIb antibodies, and then re-probed with anti-alpha tubulin antibodies.

### Gadolinium sensitivity experiments

Seeds were surface-sterilized with chlorine gas, sown on square plates (in a single row in the upper part of the plates), and stratified for 2 days in the dark at 4 °C. After stratification, seeds were germinated in a growth chamber under a 16-h light/8-h dark cycle at 21 °C, with plates kept in a vertical position. Plates containing 1/5 strength (0.86 gr/L) MS medium (Duchefa) pH = 5.7, 1% sucrose and 0.8% phytoagar, were supplemented with gadolinium (III) chloride hexahydrate (Sigma-Aldrich). Primary root length was scored after 5 or 6 days of growth with ImageJ software. Different genotypes were sown side by side on the same plate and in multiple combinations to buffer possible positional effects.

### Data availability

The authors declare that all data supporting the findings of this study are available within the manuscript and its Supplementary Files or are available from the corresponding authors upon request.

## Electronic supplementary material

Supplementary Information
