# Supplementary material for: A mechanosensitive Ca2+ channel activity is dependent on the developmental regulator DEK1
Source: Nat Commun. 2017 Oct 18;8:1009. doi: 10.1038/s41467-017-00878-w (PMC5647327; doi:10.1038/s41467-017-00878-w)
Supplement: Supplementary file 1 — Supplementary Information [file 41467_2017_878_MOESM1_ESM.pdf]

Supplementary Fig.1.

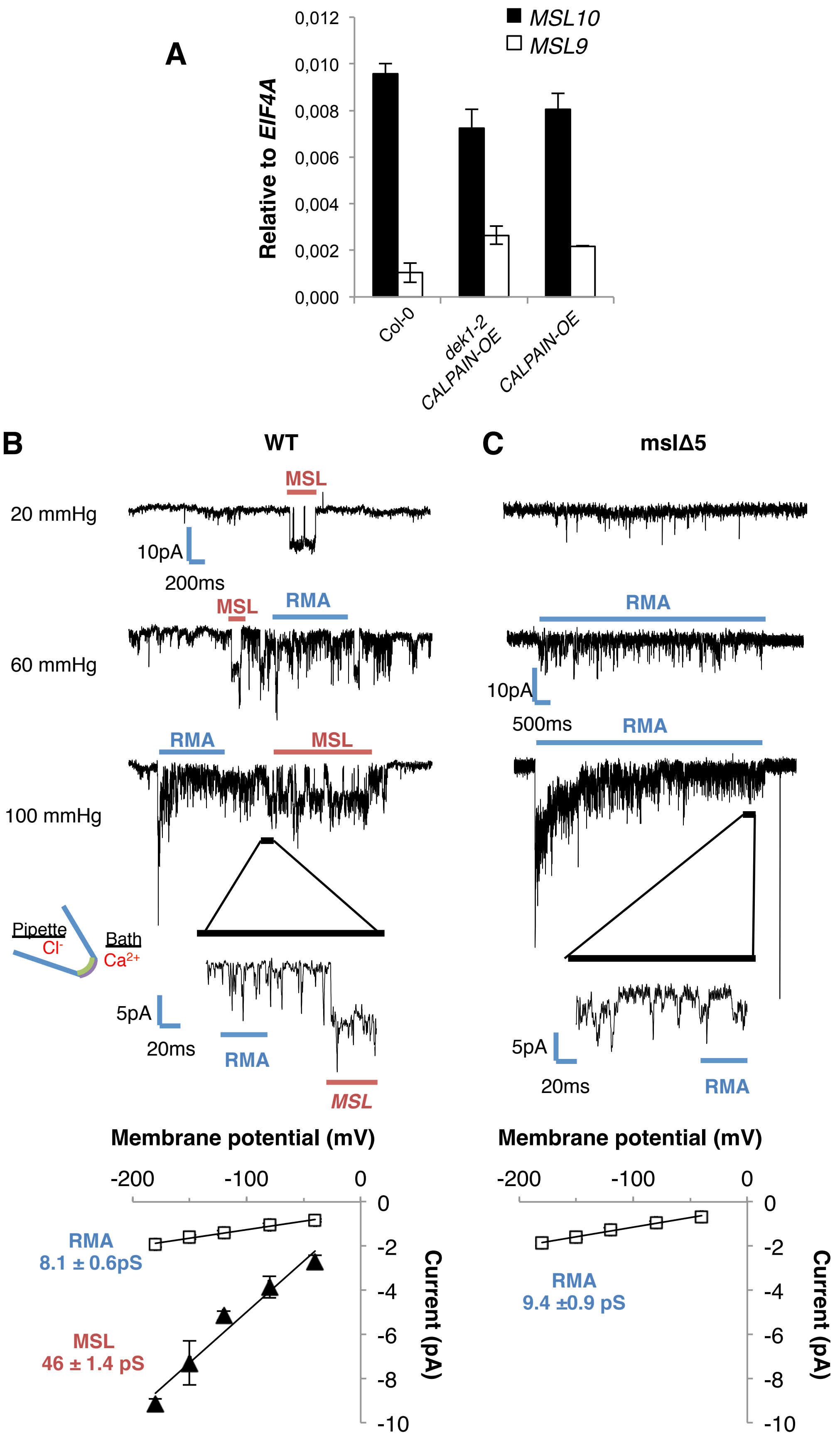

Supplementary Figure 1: Two mechanosensitive channel families permeable either to Cl^- (MSL) or Ca^{2+} (RMA) coexist at the plasma membrane

A, Confirmation of the expression of *MSL9* and *MSL10* in calli from lines used in this study. Gene expression levels were quantified by qRT-PCR and are expressed relative to expression of the *EIF4A* gene. Graph represents average of at least two independent biological replicates ± SD. **B**, In conditions favorable for both outward Cl^- and inward Ca^{2+} current recording, Col-0 membrane patches display two distinct channel activities in response to pulse pressure. Of these, the first has a small conductance (RMA) and the second has a higher conductance (MSL), as quantified in single channel I/V relationships (*bottom*) ; **C**, In the *msl* quintuple mutant *msl4;msl5;msl6;msl9;msl10* (*msl*Δ5), only the mechanically-activated current with small conductance (RMA) remains. This conductance is similar to the RMA channel activity in Col-0, as illustrated by the single channel I/V relationship (*bottom*) (n=6). For all experiments the membrane potential was held at -186 mV. Ionic conditions are described in the methods.

Supplementary Fig.2.

A

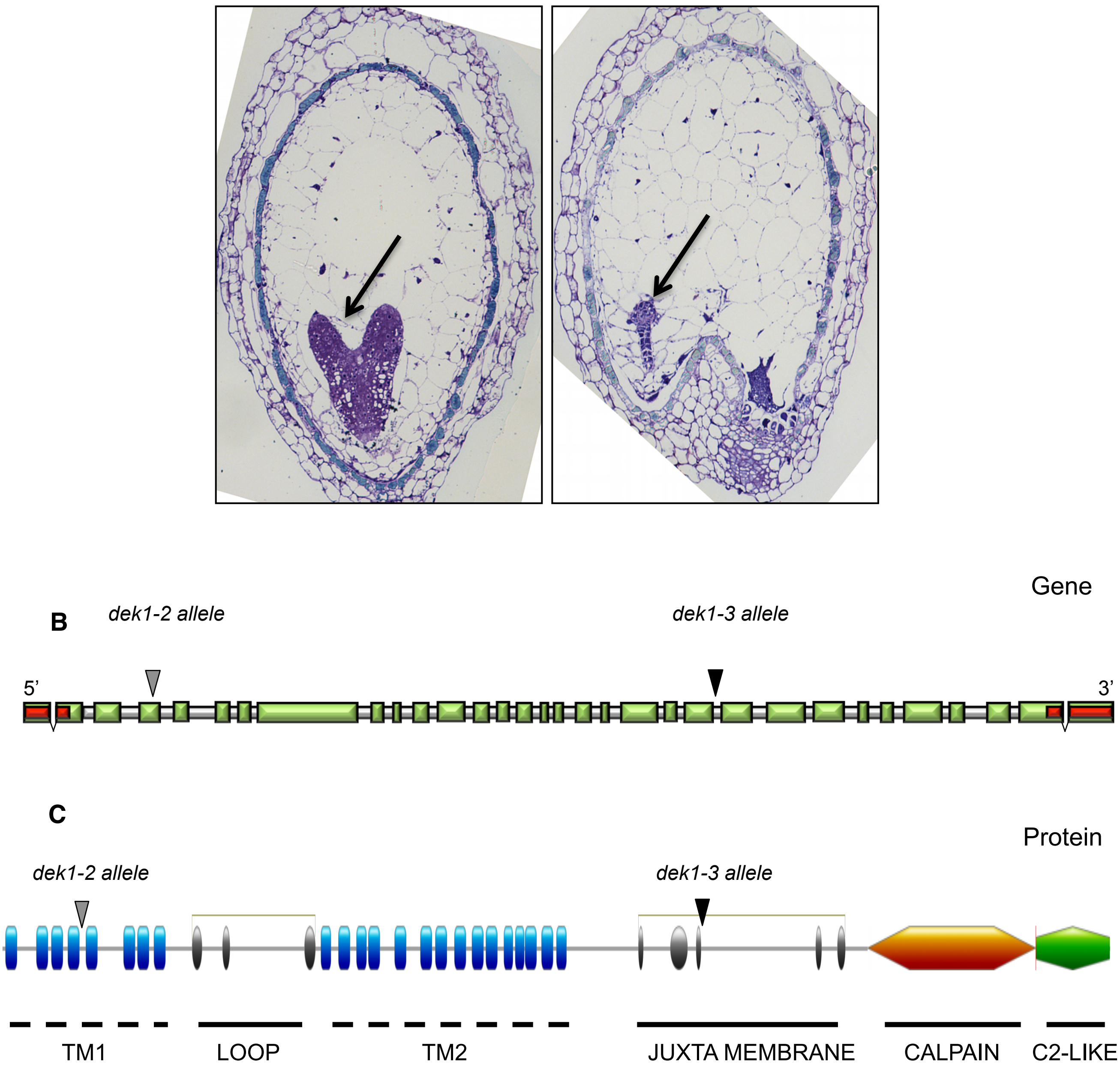

Supplementary Figure 2: Phenotype and schematic representation of *dek1* null mutant alleles used in this study.

A, Loss of DEK1 function leads to early embryo lethality (as previously reported). Comparison of seed containing a wild-type (left) and a *dek1-3* (right) homozygous mutant embryo from the progeny of self-pollinated *dek1-3* heterozygote. Seeds were both harvested 7 days after hand-pollination. Embryo is indicated with an arrow. Mutant embryos never develop past the stage shown, leading to embryo and seed lethality. *dek1-2* homozygous embryos show an identical phenotype (as previously reported). **B**, Scheme of the *DEK1* gene showing exon (green boxes) and intron (grey boxes) structure. Alternative splice sites in the 5' and 3' UTRs (red boxes) are indicated (^). **C**, Scheme of the DEK1 protein predicted domains showing predicted trans-membrane spans (TM1 and TM2 in blue), CALPAIN catalytic domain (orange hexagon), C2-like containing domain (green hexagon) and low complexity regions (grey). The position of the T-DNA insertions in *dek1-2* and *dek1-3* are shown in both gene **(B)** and protein structures **(C)**.

Supplementary Fig.3.

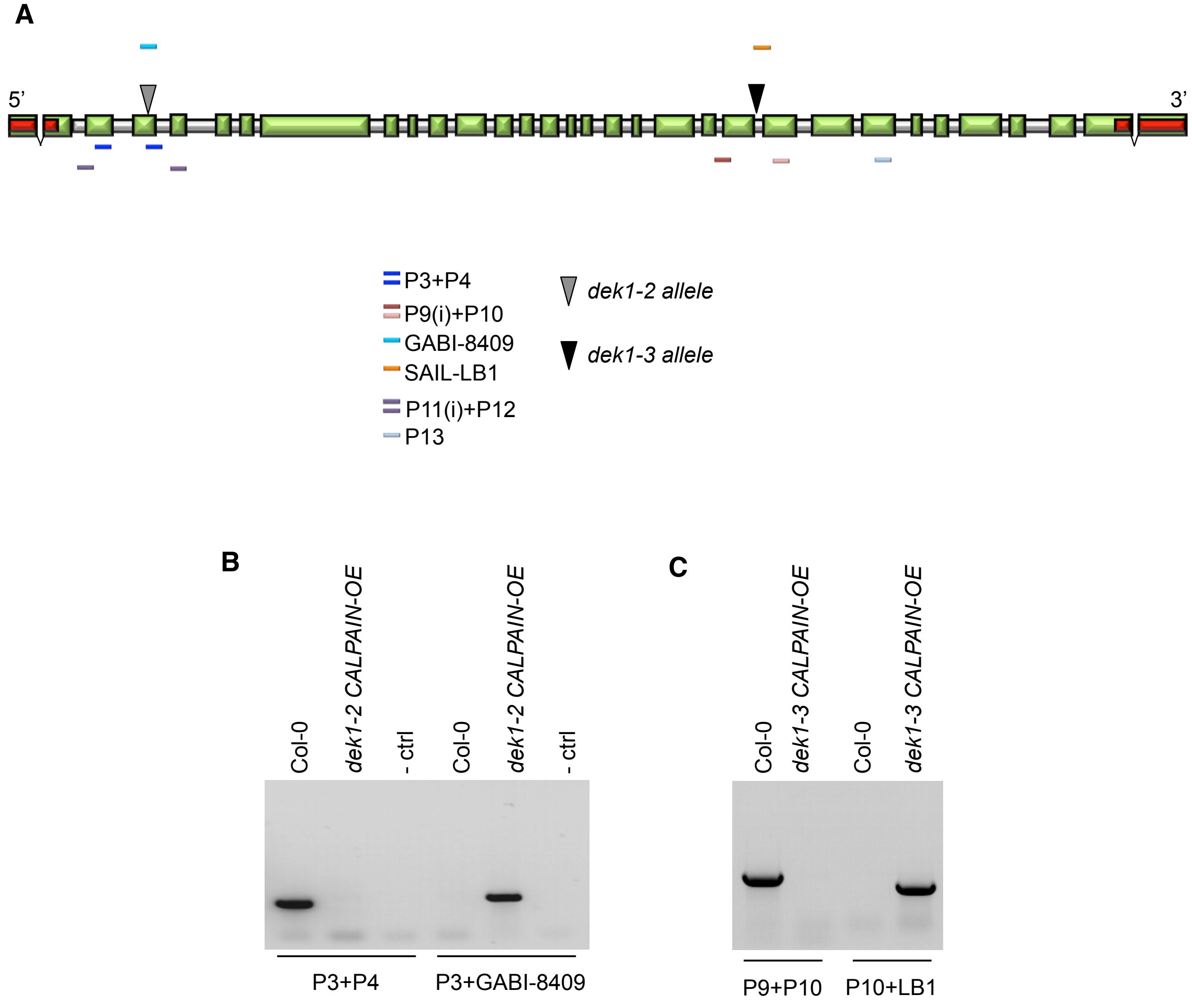

Supplementary Figure 3: Genotyping of *dek1* mutant callus material used in this study.
A, Scheme of the *DEK1* gene. Positions of primer pairs used for genotyping *dek1* mutant alleles are shown. T-DNA insertion sites for *dek1-2* and *dek1-3* mutations are shown. For experiments involving complementation of *dek1-2* with the CALPAIN domain alone, the genotyping primers P3 and P4 (which are both in exons) were used to amplify the wild-type copy of the *DEK1* gene at the *dek1-2* insertion site (as in **B**). When complementing with the full length *DEK1* cDNA, primers P11 and P12 were used to amplify the wild-type copy of the *DEK1* gene at the *dek1-2* insertion site, since this combination cannot amplify the cDNA (P11 is in an intron), and thus cannot produce an interfering band from the full length transgene. Primers situated in introns are annotated (i). To confirm the presence of the CALPAIN-encoding transgene, we used P13 combined with a primer in the *RPS5A* promoter. To confirm the presence of the full-length DEK1-encoding transgene we used P4 combined with a primer in the *RPS5A* promoter. **B,C,** Illustration of genotyping for homozygosity of the *dek1-2* and *dek1-3* mutations in *dek1-2 CALPAIN-OE* (**B**) and *dek1-3 CALPAIN-OE* (**C**) calli, respectively. Control lanes are water controls (no DNA added).

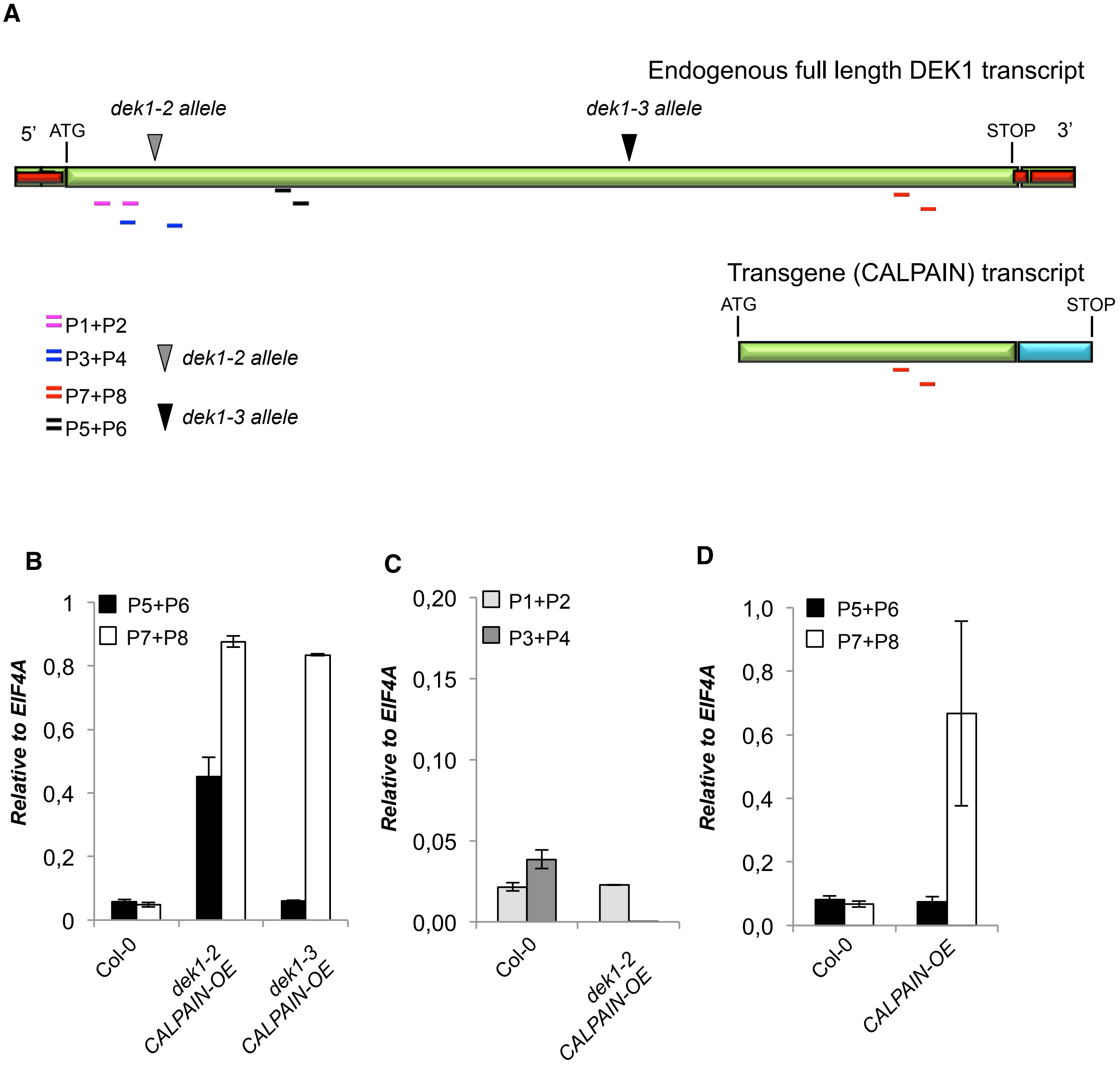

Supplementary Figure 4: Analysis of *DEK1* mRNA expression levels in callus material used in this study

A, Scheme of the *DEK1* full-length endogenous transcript and of the CALPAIN transgene transcript. Transcript CDS are drawn in green, 5' and 3' UTRs in red, tags in blue and T-DNA insertion sites for the *dek1-2* and *dek1-3* mutations are indicated by triangles pointing downwards. Positions of primer pairs used in RT-quantitative PCR analysis are shown. **B-D**, *DEK1* mRNA levels in wild-type Col-0, *dek1-2 CALPAIN-OE, dek1-3 CALPAIN-OE* and *CALPAIN-OE* calli. Gene expression levels were quantified by qRT-PCR and are expressed relative to expression of the *EIF4A* gene. Graph represents average of at least two independent biological replicates ± SD.

Supplementary Fig.5.

A

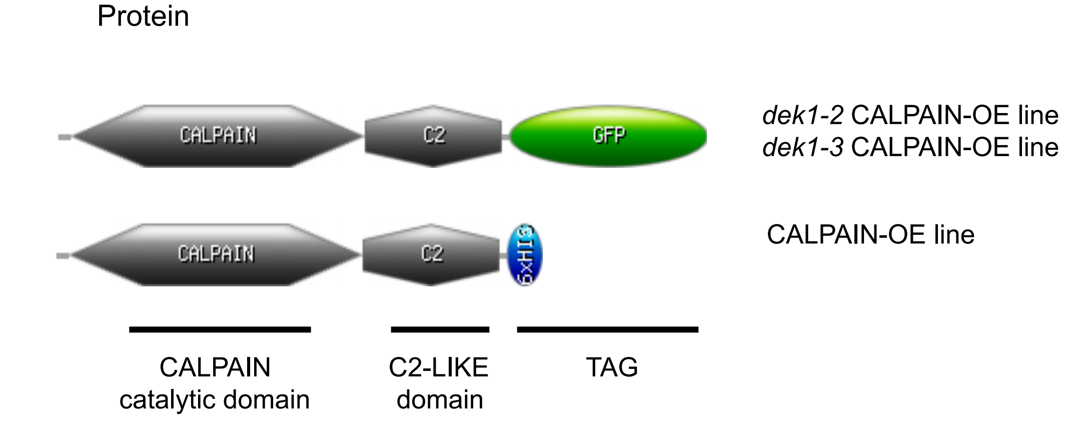

B

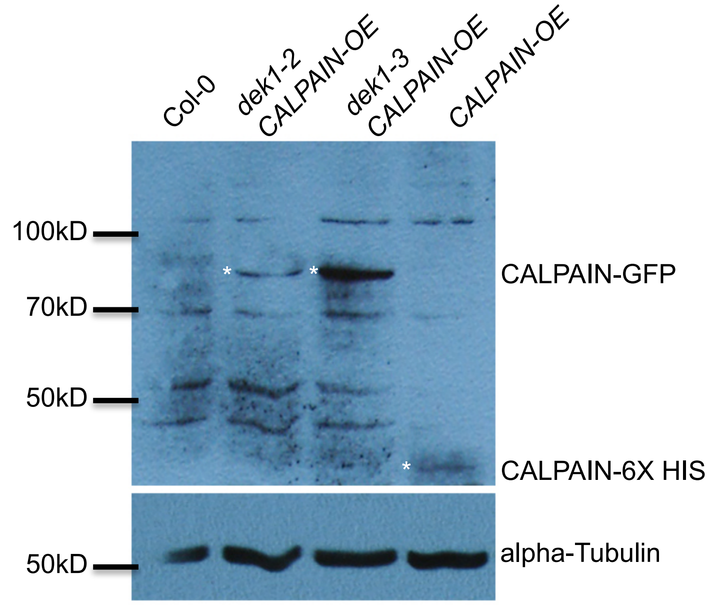

Supplementary Figure 5: Analysis of CALPAIN protein levels in callus material used in this study

A, Scheme of the transgenic constructs used for the *in pl*anta expression of the DEK1 CALPAIN domain (grey). A construct containing the CALPAIN fused to a GFP tag has been used to complement the *dek1* embryo lethal mutants, and a complementing CALPAIN fused to a 6x Histidine tag was used to transform the wild-type background. **B**, Western-blot analysis of CALPAIN expression in calli from Col-0, *dek1-2 CALPAIN-OE, dek1-3 CALPAIN-OE* and *CALPAIN-OE*. Stars = CALPAIN-GFP or CALPAIN-6x HIS. Alpha-Tubulin was used as a loading control.

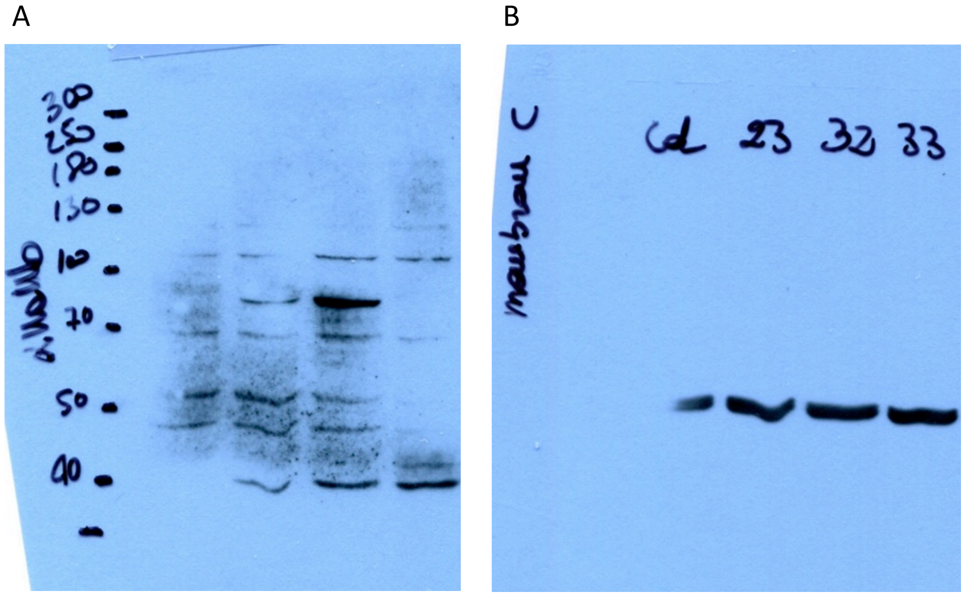

Supplementary Figure 6: Uncropped Western blots used in Supplementary Figure 5
(A) Western-blot analysis of CALPAIN expression in calli from Col-0, *dek1-2 CALPAIN-OE, dek1-3 CALPAIN-OE* and *CALPAIN-OE*. **(B)** Alpha-Tubulin was used as a loading control.

Supplementary Fig.7.

A

Line	n	Responsive	Inactivation	No inactivation/low current
Col-0	18	13	10	3
Col-0 CALPAIN-OE	19	15	13	2
dek1-2 CALPAIN-OE	25	16	2	14
dek1-3 CALPAIN-OE	21	14	10	4
Δ5	18	14	11	3
dek1-2 DEK1	17	13	6	7
dek1-3 DEK1	16	12	5	7

B

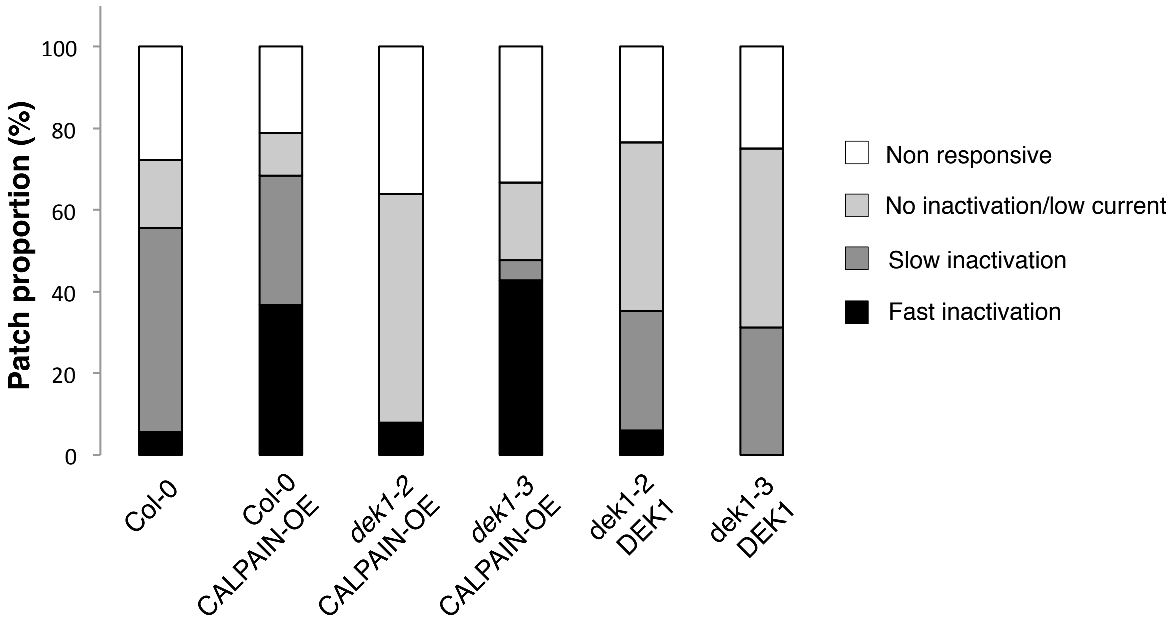

C

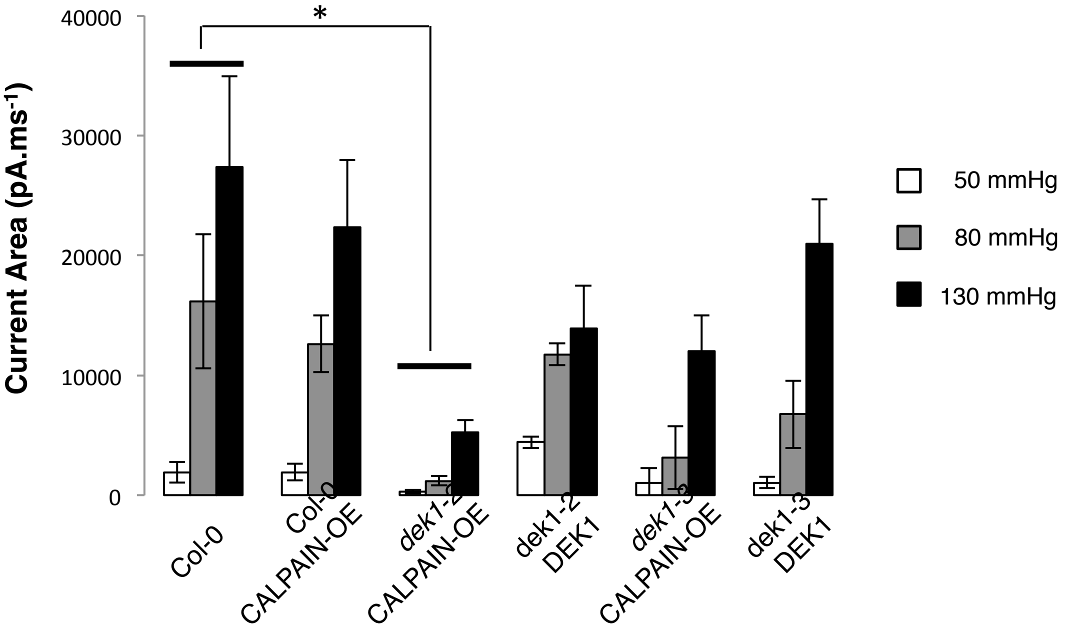

Supplementary Figure 7: Electrophysiological analysis of transgenic lines used in this study.
A, Summary of patches from all transgenic lines tested showing responsivity and inactivation kinetics. **B,** Proportions of patches showing slow and fast inactivation kinetics in response to positive pressure in different lines. **C,** Integration of macroscopic current induced by positive pressure pulses at 50 (*white bars*), 80 (*grey bars*) and 130 (*black bars*) mmHg in Col-0, Col-0 *CALPAIN-OE*, *dek1-2 CALPAIN-OE*, *dek1-3 CALPAIN-OE*, Col-0 *CALPAIN-OE*, *dek1-2 DEK1* and *dek1-3 DEK1* lines. Analyses were carried out on traces from responsive patches shown in (a and b). A rank sum test was used to identify statistically significant differences from the situation in wild-type patches (*P< 0.05).

Supplementary Fig.8.

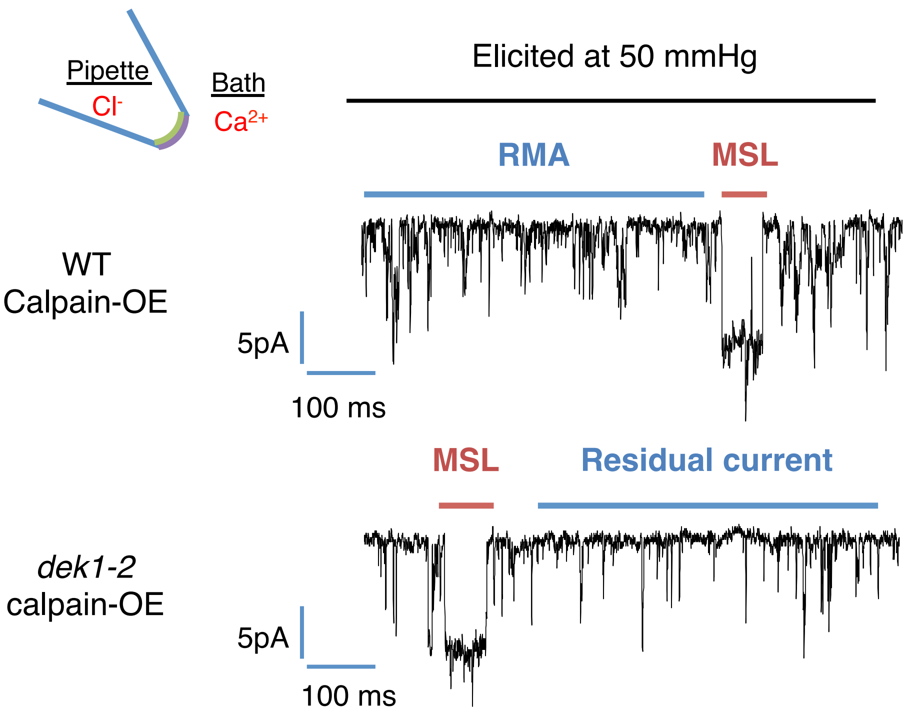

Supplementary Figure 8: MSL currents at the plasma membrane are not affected in *dek1-2 CALPAIN-OE* membranes

Representative single channel recordings in Col-0 (*top*) and *dek1-2 CALPAIN-OE* (*bottom*) membrane patches in an outside out configuration. MSL channel activities are detectable upon membrane stretching in both backgrounds. For all experiments the membrane potential was held at -186 mV. Ionic conditions are described in the methods.

Supplementary Fig.9.

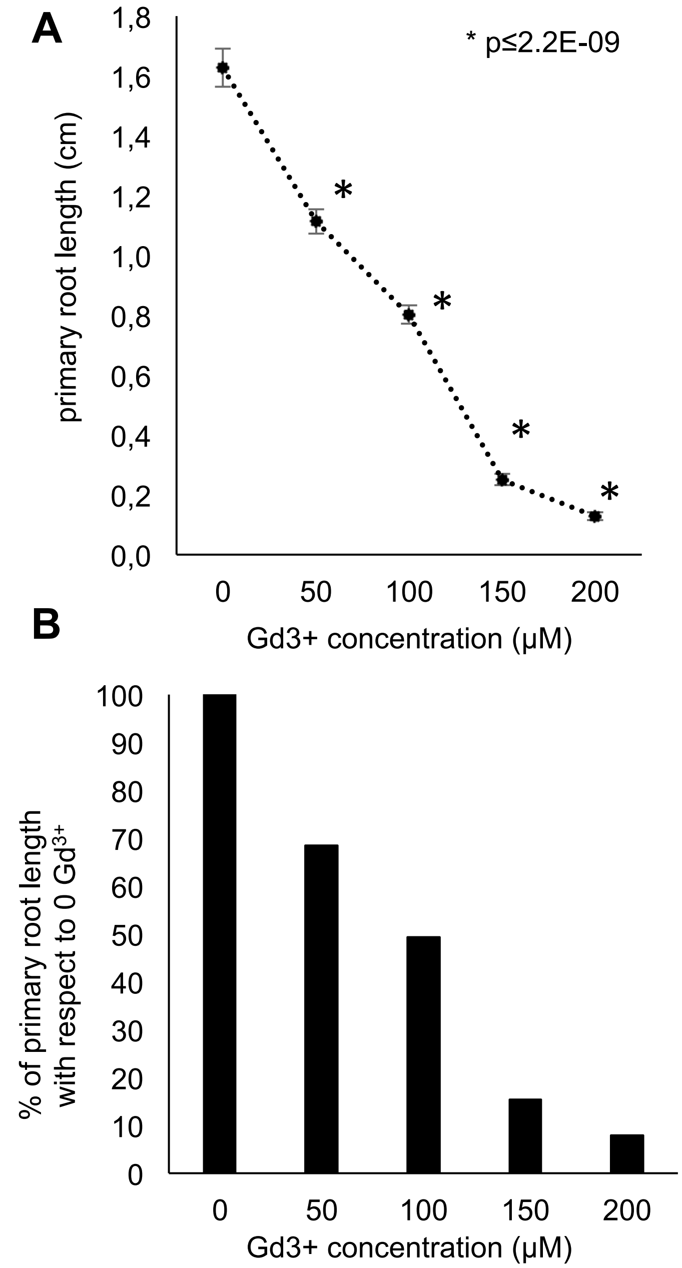

C

0 μM Gd^{3+} n=58
50 μM Gd^{3+} n=50
100 μM Gd^{3+} n=56
150 μM Gd^{3+} n=47
200 μM Gd^{3+} n=56

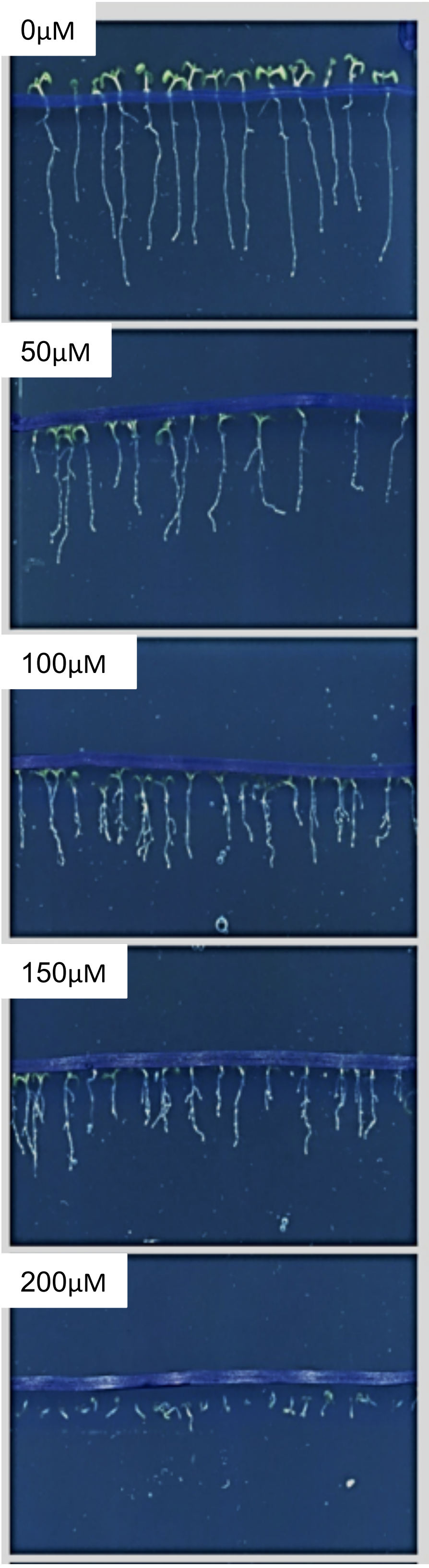

Supplementary Figure 9: Sensitivity to Gd^{3+} ions in WT plants.
Dose-effect curve for 6-day-old wild-type (Col-0) seedlings in response to different Gd3+ concentrations. **A**, average of primary root length in cm ± SE. P values are from unpaired 2-tailed T-tests between each condition and the no Gd^{3+} control. **B**, % of primary root length of Gd^{3+}-grown seedlings with respect to control (0μM Gd^{3+}). **C**, Number of seedlings analysed. **D**, representative pictures of 6-day-old seedlings grown on increasing concentrations of $Gd^{3+.}$

Supplementary Fig.10.

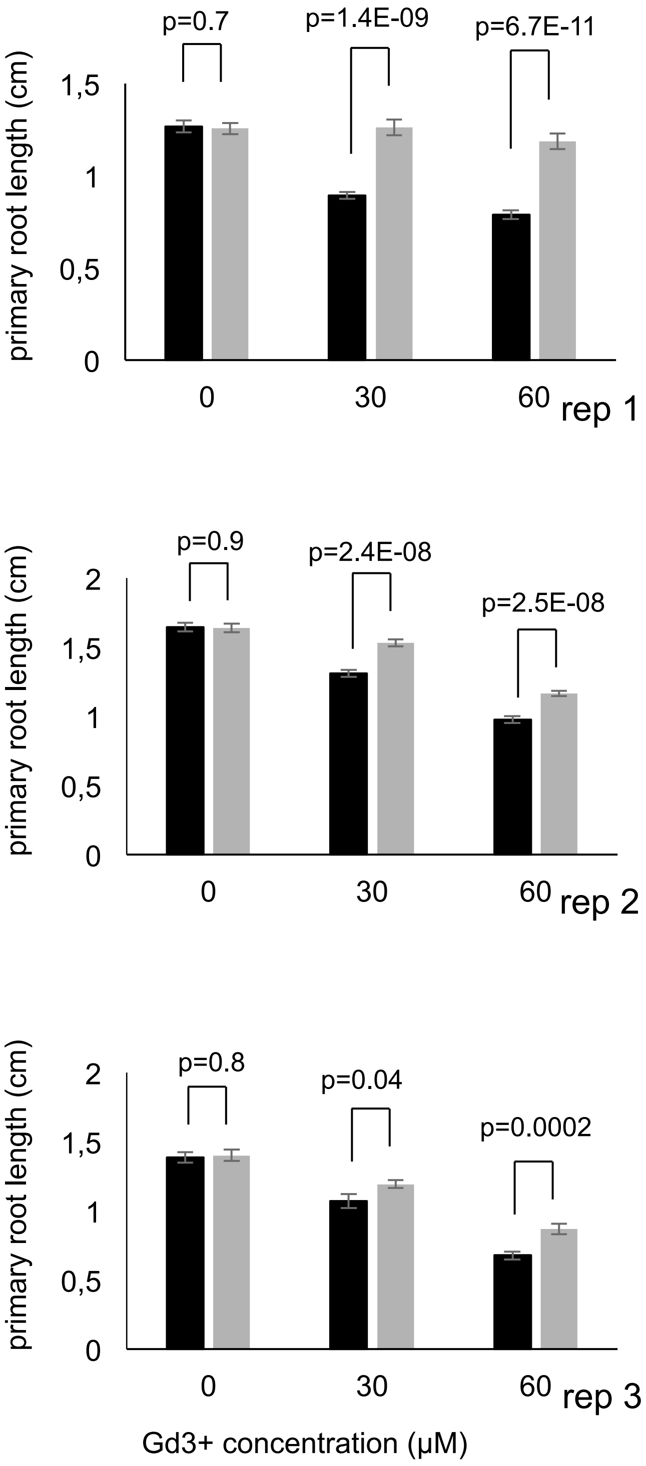

Supplementary Figure 10: Sensitivity to Gd^{3+} ions in WT and *dek1-2* plants complemented with the DEK1 CALPAIN domain.

Primary root growth of plants grown on MS or MS supplemented with 30 µM or 60 µM Gd3+. Each bar represents the average of primary root length of at least 31 samples ± SE. The experiment was repeated three times (rep 1, 2, 3). Black bars represent wild-type material and grey bars are from *dek1-2* seedlings complemented with the CALPAIN domain. P values are from two-tailed T-tests.

Supplementary Fig.11.

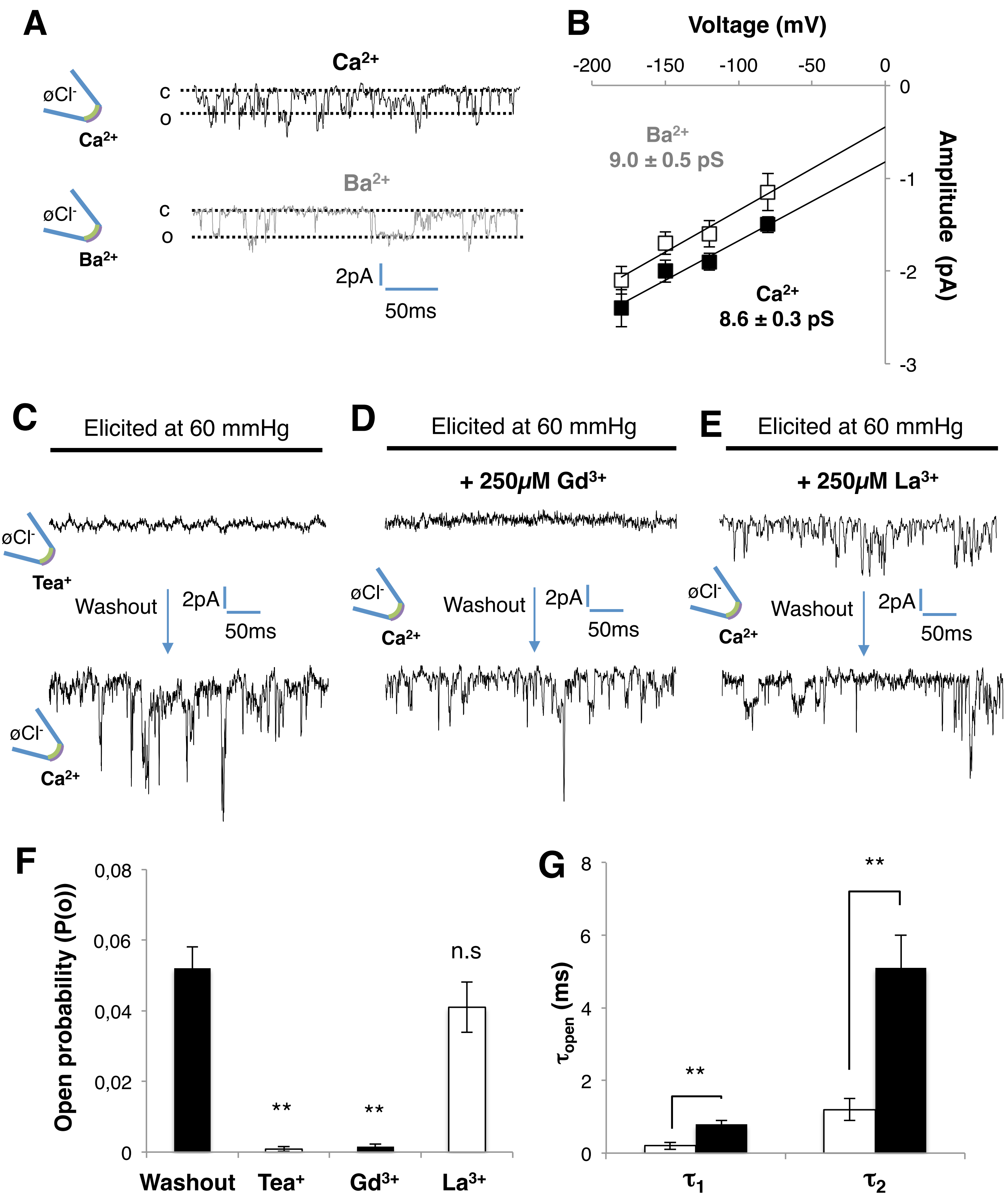

Supplementary Figure 11: Complementation of *dek1-2* mutant with the full length DEK1 protein restores RMA properties.

A, Representative single channel recordings in response to positive pressure, in an outside-out patch configuration with Ca^{2+} (top) or Ba^{2+} (bottom) as the permeant bivalent cation in the bath. The dotted lines indicate the open (O) and closed (C) channel state; **B,** Single channel I/V curves show similar conductance with permeant cations Ca^{2+} (solid square) and Ba^{2+} (open square). Values are means± SE (n=6); **C,** Representative single channel recordings showing that the RMA current is reversibly eliminated by exchanging Ca^{2+} ions with non permeant Tea^{+} ions (n=5). **D,** The mechanically-activated RMA current is inhibited by Gd^{3+} and restored after wash-out (n=4) and **E,** La^{3+} affects the open state of the RMA channel (n=3); **F,** Open probability (P(o)) in presence of different inhibitors. A paired t-test was used to compare means ($P< 0.01$); Value are means ± SE (n=5); **G,** La^{3+} ions affects the open state of the RMA channel and reduce the mean open time. Bars represent the two time constant t1 and t2 of the open state with (white bars) or without (black bars) La^{3+}. A paired t-test was used to compare means ($P< 0.01$). Values are means ± SE (open events, n≥80). For all experiments, the membrane potential was held at -196 mV. Ionic conditions are described in the methods.

**Species**	**Process**		**Reference**
A. thaliana<br>*Z. mais*	Embryo development	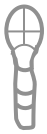	Johnson et al. 2005; Becraft et al. 2002
A. thaliana	Sepal giant cel formation	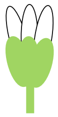	Roeder et al., 2012
A. thaliana	Trichome differentiation maintenance	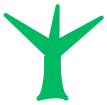	Galletti et al, 2015
A. thaliana	Pavement cell development	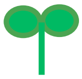	Galletti et al, 2015
Z. mais<br>*A. thaliana*	Aleurone formation	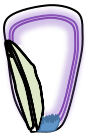	Lid et al., 2002; Lid et. al. 2005, Becraft et al., 2002
N. benthamiana	Proliferation control	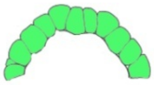	Ahn et al., 2004
P. patens	Orientation of division planes	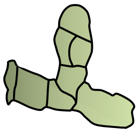	Perroud et al., 2014

Supplementary Figure 12: Summary of the developmental processes involving the *DEK1* gene.

Supplementary Table 1

Primers

Name	Sequence	AGI code	Description/ Purpose
P1	CCCTATTGCAATCTTGATAGGATG	AT1G55350	qRT-PCR
P2	GCCCAATGATATGTCGATCC	AT1G55350	qRT-PCR
P3	ATCATGCTTTGGTGGAGGAC	AT1G55350	qRT-PCR/ Genotyping
P4	CAACGCGATTGCTGATACAC	AT1G55350	qRT-PCR/ Genotyping
P5	CTTGAGAGAAGGTTTTCGGAG	AT1G55350	qRT-PCR
P6	CCTGTTCGAGTTAGATTGTCAG	AT1G55350	qRT-PCR
P7	AAACAAGAGGGGTTCTTACTTGG	AT1G55350	qRT-PCR
P8	TTCGAATCTGAACAAGTCTGTGC	AT1G55350	qRT-PCR
P9	TGGTTTGACGAAGCTGAACTC	AT1G55350 (intron)	Genotyping
P10	CTTCACTTAAGCATTTCCCCC	AT1G55350	Genotyping
P11	GTTGTTTTGTCATATATGGCGCTT	AT1G55350 (intron)	Genotyping
P12	AAGCAAATTTATATGCCCTCCTTA	AT1G55350	Genotyping
P13	TTGCTTATCACCTCGTGCAG	AT1G55350	Genotyping
MSL9L	TCGCTAAGCCAAACCACAAT	AT5G19520	qRT-PCR
MSL9R	TTCTTCCTTTATCATGAACCTCAA	AT5G19520	qRT-PCR
MSL10L	TGTGTTGTTGACGGTGTCG	AT5G12080	qRT-PCR
MSL10R	TCAAGAACACTGTCGTTAAGAGATTC	AT5G12080	qRT-PCR
pRPS5a	AGACGGCAGAGAAAACGAGA	AT3G11940 (promoter)	Genotyping
GABI8409	ATATTGACCATCATACTCATTGC		Genotyping
SAIL-LB1	GCCTTTTCAGAAATGGATAAATAGCCTTGCTTCC		Genotyping
